# Factors Related to Social Support in Neurological and Mental Disorders

**DOI:** 10.1371/journal.pone.0149356

**Published:** 2016-02-22

**Authors:** Kaloyan Kamenov, Maria Cabello, Francisco Félix Caballero, Alarcos Cieza, Carla Sabariego, Alberto Raggi, Marta Anczewska, Tuuli Pitkänen, Jose Luis Ayuso-Mateos

**Affiliations:** 1 Instituto de Salud Carlos III, Centro Investigación Biomédica en Red, CIBER, Madrid, Spain; 2 Faculty of Social and Human Sciences, School of Psychology, University of Southampton, Southampton, United Kingdom; 3 Department of Medical Informatics, Biometry and Epidemiology-IBE, Chair for Public Health and Health Services, Research, Research Unit for Biopsychosocial Health, Ludwig-Maximilians-University (LMU), Munich, Germany; 4 Swiss Paraplegic Research, Nottwil, Switzerland; 5 Neurological Institute "C. Besta" IRCCS Foundation, Neurology, Public Health and Disability Unit, Milan, Italy; 6 Department of Psychiatry, Institute of Psychiatry and Neurology, Warsaw, Poland; 7 A-Clinic Foundation, Helsinki, Finland; 8 Department of Psychiatry, Universidad Autónoma de Madrid, Madrid, Spain; 9 Instituto de investigación de la Princesa, (IIS-IP), Hospital Universitario de la Princesa, Madrid, Spain; Penn State College of Medicine, UNITED STATES

## Abstract

Despite the huge body of research on social support, literature has been primarily focused on its beneficial role for both physical and mental health. It is still unclear why people with mental and neurological disorders experience low levels of social support. The main objective of this study was to explore what are the strongest factors related to social support and how do they interact with each other in neuropsychiatric disorders. The study used cross-sectional data from 722 persons suffering from dementia, depression, epilepsy, migraine, multiple sclerosis, Parkinson's disease, schizophrenia, stroke, and substance use disorders. Multiple linear regressions showed that disability was the strongest factor for social support. Extraversion and agreeableness were significant personality variables, but when the interaction terms between personality traits and disability were included, disability remained the only significant variable. Moreover, level of disability mediated the relationship between personality (extraversion and agreeableness) and level of social support. Moderation analysis revealed that people that had mental disorders experienced lower levels of support when being highly disabled compared to people with neurological disorders. Unlike previous literature, focused on increasing social support as the origin of improving disability, this study suggested that interventions improving day-to-day functioning or maladaptive personality styles might also have an effect on the way people perceive social support. Future longitudinal research, however, is warranted to explore causality.

## Introduction

The concept of social support varies from objective social life (group memberships, family, spouse, etc.) to subjective experience (e.g., emotional support, loneliness). Research on social support has primarily focused on its protective role against the negative effects of stressful life events or its positive impact on psychological well-being, mortality and disease severity [1–5]. Lower social support has been associated with poor treatment outcomes [6] and has predicted higher health care utilization in mental disorders [7]. Enhancement of social support has been recommended as an important part of the treatment for mental and neurological disorders [8].

Some studies agree that people with neuropsychiatric disorders have smaller and poor quality social networks [5, 9–11]. Literature so far has investigated certain personality, disease and disability related factors as potential variables explaining this phenomenon. On one hand, studies in some neuropsychiatric disorders have shown associations between personality types and level of social support [12]. More specifically, three of the “Big Five” personality traits—extraversion, agreeableness and conscientiousness have shown positive correlation with variables related to social support in depression, whereas a negative correlation has been found for neuroticism and openness [13]. Furthermore, association between gender and social support is evident in epilepsy [14], whereas quality of life has shown significant relation with social support in schizophrenia [15]. However, many personality related variables like resilience, or socio-demographic factors such as level of education, marital status or age, are still under-researched as potential factors for social support in number of neuropsychiatric conditions.

Another line in research postulates that certain disability or disease-specific factors might be also associated with social support. High functional disability has been associated with lower social support in depression [16] and migraine [17]. Social support has also moderated the relationship between depressive symptoms and functional disability. Adults with higher functional impairment benefited less from increased social support in the context of higher symptom severity in depression [7, 18, 19].

Despite all published studies so far, there are still four issues that remain unresolved. First, literature exploring social support as dependent variable is scarce. Many relevant personality disability and disease-specific factors for social support are under-researched. Although some evidence mainly on the independent effect of personality and disability in neurological and mental disorders exists, it is somehow limited and focuses mainly on one single condition. Second, we still do not know which the most important factor for social support is. Personality, clinical and disability factors included in a single model have not been studied. Third, no study has analyzed whether the interaction effect of personality and health condition related factors is stronger than these factors alone. Moreover, no mediation effects have been explored. Finally, social support in mental and neurological disorders has not been compared so far. It is still unknown whether people with mental disorders experience different levels of social support compared to neurological disorder when the level of functioning is included in the interaction.

In attempt to address all these issues and investigate why people with neuropsychiatric disorders experience poor social support, this study aimed to explore 1) what are the strongest factors related to social support in neuropsychiatric disorders, 2), whether there is a specific interaction between the most important personality and health related factors for social support 3) whether the level of disability mediates the relationship between personality and social support and 4) whether type of health condition (mental or neurological) is a moderator between level of disability and social support.

## Methods

### Procedure

This cross-sectional study was carried out using data from the European Union (EU) funded coordinated project “Psychosocial fActors Relevant to brAin DISorders in Europe (PARADISE)” [20], (http://paradiseproject.eu/). The study was conducted according to the ethical principles of the European Commission (EC) Research Ethics Committee and approved by the Ethics Committee of the Ludwig-Maximilian University, Munich, Germany, as a coordinating center, and by the local Ethics Committees of each recruiting institution.

### Sample

Data was gathered from 722 participants: patients with stroke (n = 80), multiple sclerosis (n = 80), epilepsy (n = 80), migraine (n = 80) and Parkinson Disease (n = 80) were recruited at the Neurological Institute Carlo Besta IRCCS Foundation in Milan, Italy, while patients with dementia (n = 80) and schizophrenia (n = 81) were recruited at the Institute of Psychiatry and Neurology in Warsaw, Poland; finally, patients with depressive disorders (n = 81) and substance-use disorders (n = 80) were recruited, respectively, at La Princesa University Hospital in Madrid, Spain, and the Järvenpää Addiction Hospital in Järvenpää, Finland.

The participants were referred by their main health professionals. All the interviews were face to face and conducted by mental health professionals trained to use the PARADISE protocol. The inclusion criteria were broad in order to catch the full range of psychosocial difficulties experienced by individuals: ≥ 18 years of age and a main diagnosis established according to ICD-10 [21]. The majority of patients had comorbid disorders, some of which among the ones included in this study, but this was not a reason for exclusion. Every patient was listed in one of the nine groups of disorders according to his primary diagnosis. Participants were informed of the rationale of the study and asked to sign an informed consent form. For sufferers of dementia not able to provide consent, caregivers were asked to do it and participate in the interviews as proxies.

### Measures

Since a global score was obtained for each of the scales considered in the study, Confirmatory Factor Analyses (CFA) were conducted in order to obtain evidence for one-factor solutions and to assume unidimensionality before obtaining these global scores. Goodness-of-fit of the proposed model was evaluated according to the standard recommendations [22, 23]. Values of the Comparative Fit Index (CFI) above 0.90 were considered to represent an adequate fit; values of Root Mean Square Error of Approximation (RMSEA) less than 0.08 indicated a good fit [24].

Social support was measured using the Oslo 3-item Social Support Scale [25], covering three items on primary support group (“*How many people are you so close to that you can count on them if you have great personal problems*?”), interest and concern shown by others (“*How much interest and concern do people show in what you do*?”), and ease of obtaining practical help (“*How easy is it to get practical help from neighbors if you should need it*?*”)*. The instrument has been used in various studies thus proving its feasibility [26]. A global score was created by adding up the raw scores, ranging from 3 to 14 with higher scores indicating higher social support. Since this instrument comprised three items, the overall fit of a one-factor model could not be ascertained because there were no degrees of freedom. However, the three items representing social support had significant loadings on the latent construct (factor loadings higher than .45). This finding suggests that the three items of the scale represent social support and a global score can be obtained from these three items.

Disability was assessed by World Health Organization Disability Assessment Schedule short-form 12 (WHODAS-12) [27]. The questionnaire measures global disability and is suitable for epidemiological studies and outcome assessments. Its psychometric properties have been measured in dozens of studies in diverse cross-cultural settings [28, 29]. The total score ranges between 0 and 100 as higher scores indicate higher levels of disability, i.e., lower levels of functioning. A CFA was performed over the WHODAS items on the overall sample and evidence for a one-factor solution was found (CFI = 0.961, RMSEA = 0.067).

Another instrument developed by WHO (EUROHIS-QOL 8-item index) was used to measure the quality of life [30]. It is a shortened version of the World Health Organization Quality of Life Instrument-Abbreviated Version (WHOQOL-BREF). It was designed as a short and concise instrument of eight items reporting subjective well-being and satisfaction with different life aspects. Psychological, social, physical and environmental domains were assessed each with two items. The overall quality of life score is formed by the sum of the scores of the eight items, with higher scores indicating better quality of life. EUROHIS-QOL has also shown adequate cross-cultural validity [31]. A CFA was conducted in order to test the unidimensionality of the eight items of the EUROHIS-QOL. The CFA performed on the overall sample presented acceptable values in the goodness-of-fit indices: CFI = 0.978 and RMSEA = 0.063.

Resilience was assessed with the Brief Resilient Coping Scale [32]. The questionnaire was designed to capture tendencies of individuals to cope with stress. The instrument captures the following themes: tenacity, optimism, creativity, an aggressive approach to problem solving, and commitment to positive growth from difficult situations. The questionnaire has 4 items giving a range of 4–20 with higher scores indicating higher resilience. The CFA performed on the overall sample provided support for a one-factor solution: CFI = 0.983 and RMSEA = 0.025.

Personality style was measured with the short version of the Big Five Inventory (BFI), BFI-10 [33, 34]. The instrument is a self-report inventory designed to measure the Big Five personality dimensions—conscientiousness, agreeableness, neuroticism, extraversion and openness. It consists of 10 items in total (2 for each personality trait) and has been previously used in a number of studies [35].

Severity of symptoms was a composite variable. The severity score of each disease, measured by a previously used instrument, was combined in one new variable. The new severity of symptoms index variable was created according to three previously established cut-off points (mild, moderate and severe), amalgamating scores for depression (Hamilton Depression Rating Scale (HAM-D; [36]); dementia (Mini-Mental State Examination (MMSE; [37]); migraine (The Migraine Disability Assessment Test (MIDAS); [38]); multiple sclerosis (Expanded Disability Status Scale (EDSS); [39, 40]; Parkinson’s disease (Hoehn & Yahr Staging scale; [41]); schizophrenia (Brief Psychiatric Rating Scale (BPRS); [42]); stroke (National Institutes of Health Stroke Scale (NIHSS); [43]) and alcohol dependence (Alcohol Dependence Scale (ADS); [44]. Data on substance use disorders was collected with the ‘Severity of Dependence Scale’ and the “Alcohol Dependence Scale (ADS)”. Our intention was to use the collected data from the ‘Severity of Dependence Scale’, but due to the large number of missing data we report here only the alcohol dependent participants, filled out the ADS questionnaire.

Comorbidity was assessed with Self-reported Comorbidities Questionnaire (SCQ) [45]. The summary score represents the addition up to three points derived from each reported health condition: a point for its presence, point if treatment is received, and point if it causes decrements in functioning. Demographic variables such as gender, age, education and marital status were included in the analysis.

### Statistical analysis

First, descriptive statistics were obtained, including summary of the socio-demographic data of the participants and means and standard deviations (SDs) of the survey scale scores. Second, a multiple linear regression analysis was conducted to explore the relationships between the two groups of independent variables considered: personality related variables (personality type, resilience, quality of life, gender, age, education and marital status) and health condition related factors (disability, severity of symptoms, and comorbidity) and the score of social support. All the independent variables were introduced simultaneously in the model because we were exploring potential predictors of social support rather than comparing them or introducing previously established models. The ordinary least squares (OLS) estimation was employed; the use of OLS trades robustness for some improvement in efficiency [46] and has been shown to yield the best fit of data [47]. Beta coefficients were reported and can be interpreted as change in the outcome (in standard deviations) per standard deviation change in the predictors; they were used to assess which variables had the highest association with the outcome variable. 95% confidence intervals (CI) were reported.

We assessed three regression models. The first model included all the independent variables. In the second model, dummy variables for type of health condition (dementia, epilepsy, migraine, multiple sclerosis, Parkinson, schizophrenia, stroke and substance use disorders) were added to check for their association with social support and to control for their potential effect. In a third model, the interaction terms between the most significant personality and health condition related factors were added to model 2. Since we entered multiple independent variables, the presence of multicollinearity was assessed by means of the Variance Inflation Factor (VIF). Values below 5 have been considered adequate [48].

Mediation and moderation analyses were conducted, respectively, to: 1) assess if disability mediates the relationship between the personality traits which were found significant on the previous regression models and social support; 2) assess if the relationship between disability and social support is moderated by the type of health condition (mental vs. neurological). In each mediation analysis, two linear regression models were considered: one for the relationship between the independent and the mediating variable, and another for the relationship between the mediating and the dependent variable. In the moderation analysis, the interaction term (the independent multiplied by the moderating variable) was calculated, and then by means of a "simple slope" analysis was assessed whether the gradient of both lines for mental and neurological conditions differed from 0. All the health conditions were merged into two groups: mental (depression, substance use disorders, schizophrenia) and neurological health conditions (dementia, epilepsy, migraine, multiple sclerosis, Parkinson’s disease and stroke).

SPSS 21.0 software [49] was used to analyse socio-demographic data and to conduct the regression models. For testing mediation, online MedGraph programme was used to compute the correlations and regressions [50]. We introduced the values of the unstandardized regression coefficients and standard errors of path *a* and path *b* to MedGraph. Path *a* represents the unstandardized regression coefficient for the path from the independent variable (personality type) to mediating variable (disability); path *b* is the coefficient for the path from the mediating variable (disability) to the dependent variable (social support) controlling for the independent variable. Sobel’s z-score and *p*-values for significance were then computed. Indirect to Total ratio was also computed, reporting the estimated size of the indirect effect in relation to the total effect. The indirect to total ratio explains the amount of variance in the relationship between the independent and the dependent variables which is due to the mediation variable. Values fall between .00 and 1.00, with higher values indicating higher relative indirect effects [51]. For the moderation analysis the process was identical and conducted by means of the online ModGraph programme [52].

## Results

### Descriptive statistics

Demographics, socio-economic and clinical characteristics are presented in Table 1. The mean age of the total sample was 51.29 years (SD = 14.25). There were a similar proportion of men and women, and most of the participants had at least completed high school. A greater number of people with epilepsy, migraine and multiple sclerosis were still working despite their condition. In some conditions like schizophrenia and substance use disorders people were not employed although they were younger. As for the clinical characteristics, people with depression and substance use disorders showed the greatest number of comorbid conditions. Table 1 shows descriptively that the level of social support and quality of life among the conditions was relatively equal, but people suffering from depression, schizophrenia and substance use disorders had a higher result on the disability scale.

**Table 1:** Demographic, socio-economic and clinical characteristics of the sample.

	Epilepsy	Migraine	Multiple Sclerosis	Parkinson	Stroke	Dementia	Depression	Schizophrenia	Substance Use
N	80	80	80	80	80	80	81	81	80
Age (years)									
Mean (SD)	41.23 (11.99)	44.54 (12.12)	41.03 (8.74)	61.24 (10.45)	59.84 (14.36)	81.03 (5.49)	54.81 (14.73)	38.38 (14.03)	39.56 (13.15)
Gender (%)									
Female	50.0	86.3	65.0	40.0	43.8	78.8	82.7	53.1	37.5
Marital status (N)									
Married or in a relationship	42	53	50	65	62	25	37	10	29
Not married	38	27	30	15	18	55	44	71	51
Less than primary school	0.0	0.0	1.3	1.3	0.0	3.8	17.3	0.0	3.8
Primary school completed	1.3	1.3	1.3	13.8	22.5	18.8	16.0	4.9	35.0
Education (%)									
Secondary school completed	28.8	18.8	23.8	26.3	22.5	3.8	11.1	4.9	32.5
High school completed	50.0	48.8	51.3	43.8	40.0	40.0	17.3	44.4	17.5
University or postgraduate degree completed	20.0	31.3	22.5	15.1	10.0	33.8	38.3	45.7	11.3
Working sample (%)	66.3	67.5	72.5	33.8	25.0	0.0	27.2	8.6	6.3
Disease duration (years)									
Mean (SD)	18.67 (12.32)	21.13 (14.60)	7.66 (6.94)	6.26 (4.40)	4.00 (6.48)	3.69 (2.70)	12.63 (11.57)	13.03 (11.83)	12.16 (8.67)
Comorbidity score (SCQ score)									
Mean (SD)	2.36 (2.97)	2.36 (2.55)	1.18 (2.17)	3.00 (2.63)	5.87 (4.87)	5.86 (4.17)	12.56 (5.09)	2.72 (3.14)	8.84 (4.76)
Social Support (OSS)									
Mean (SD)	10.92 (1.32)	10.05 (2.30)	10.46 (2.05)	10.02 (1.99)	10.32 (1.49)	9.45 (2.18)	10.09 (2.55)	8.93 (2.63)	9.64 (2.04)
Quality of Life (WHOQOL)									
Mean (SD)	14.02 (2.31)	14.30 (2.61)	13.43 (2.47)	14.43 (2.22)	13.80 (2.48)	15.27 (2.12)	16.41 (3.05)	15.15 (2.81)	14.40 (2.67)
Extraversion									
Mean (SD)	2.98 (0.88)	3.02 (1.09)	3.28 (1.01)	2.90 (0.94)	3.05 (0.88)	3.14 (0.84)	3.16 (1.33)	2.89 (1.03)	3.11 (1.02)
Agreeableness									
Mean (SD)	3.57 (0.71)	3.66 (0.89)	3.80 (0.90)	4.01 (0.89)	3.55 (0.76)	3.76 (0.92)	4.00 (1.12)	3.33 (1.12)	3.24 (0.84)
Disability (WHODAS)									
Mean (SD)	10.88 (10.45)	21.56 (13.13)	15.75 (15.06)	18.65 (15.85)	19.17 (15.43)	30.93 (18.13)	42.40 (18.05)	35.05 (22.06)	39.62 (20.25)

### Factors, associated with social support

The models of regression conducted are shown in Table 2. In the first model disability, extraversion, agreeableness, gender, age and comorbidity were significantly related with social support. In the second model the type of health condition was added, but the increase in the variance explained was not significant (ΔR^2^ = 0.016, p = .21). The results revealed that disability (ß = .19, = .002), agreeableness (ß = .17, p < .001) and extraversion (ß = .15, p = .001) were the strongest factors for social support. Gender, age and comorbidity were significant in Model 1, but when the health conditions were introduced to the model, only gender remained significant (ß = .10, p = .027), meaning that men experienced higher social support than women. Quality of life, marital status, level of education and type of health condition were not significant factors. All the variables considered in the models had an associated VIF value lower than 5 (mean VIF of 2.20), indicating that the assumption of no perfect multicollinearity can be assumed to conduct the regression model. A post hoc power analysis was performed for the total sample of 722 individuals, considering a significance level of .05, and obtaining a statistical power higher than .90.

**Table 2:** Multiple linear regression analysis assessing the relationships between the independent variables considered and social support.

Variables		Model 1			Model 2			Model 3	
	B (95% *CI*)	*p*	|ß|	B (95% *CI*)	*p*	|ß|	B (95% *CI*)	*p*	|ß|
Severity of symptoms (*Ref*. = *Mild*)									
Moderate	0.22 (-0.21, 0.66)	.31	0.05	0.19 (-0.28, 0.66)	.42	0.04	0.21 (-0.26, 0.68)	.38	0.05
Severe	0.55 (0.05, 1.04)	**.029**	0.11	0.42 (0.16, 1.01)	.15	0.09	0.42 (0.16, 0.99)	.15	0.09
Disability	-0.03 (-0.04, -0.01)	<.**001**	0.24	-0.02 (-0.03, -0.01)	**.002**	0.19	-0.07 (-0.11, -0.03)	**.001**	0.66
Resilience	0.01 (-0.07, 0.06)	.*90*	0.01	0.02 (-0.04, 0.09)	.49	0.03	0.02 (-0.05, 0.08)	.60	0.03
Quality of Life	-0.07 (-0.14, 0.01)	.*08*	0.08	0.06 (-0.13, 0.02)	.13	0.07	-0.06 (-0.14, 0.01)	.11	0.07
Extraversion	0.34 (0.16, 0.52)	<**.001**	0.16	0.32 (0.13, 0.49)	**.001**	0.15	0.01 (-0.30, 0.27)	.92	0.01
Openness	-0.01 (-0.19, 0.17)	.*91*	0.01	0.01 (-0.18, 0.19)	.95	0.01	-0.02 (-0.20, 0.16)	.86	0.01
Neuroticism	-0.11 (-0.29, 0.08)	.*26*	0.05	-0.10 (-0.29, 0.09)	.29	0.05	-0.08 (-0.27, 0.10)	.37	0.04
Conscientiousness	0.19 (-0.01, 0.39)	.*07*	0.08	0.20 (-0.02, 0.41)	.07	0.08	0.21 (-0.01, 0.42)	.05	0.09
Agreeableness	0.36 (0.16, 0.55)	<.**001**	0.16	0.38 (0.18, 0.59)	<**.001**	0.17	0.26 (-0.06, 0.59)	.11	0.11
Disability*Agreeableness							0.004 (-0.005, 0.01)	.35	0.16
Disability*Extraversion							0.01 (0.004, 0.02)	**.004**	0.37
***Controlling variables***									
Marital status	-0.21 (-0.57, 0.15)	.25	0.05	-0.17 (-0.56, 0.21)	.37	0.04	-0.14 (-0.53, 0.24)	.47	0.03
Education	-0.01 (-0.18, 0.17)	.96	0.01	0.01 (-0.17, 0.19)	.92	0.01	0.003 (-0.18, 0.18)	.99	0.01
Gender	0.50 (0.13, 0.86)	**.008**	0.11	0.44 (-0.05, 0.83)	**.027**	0.10	0.45 (0.06, 0.84)	**.024**	0.10
Age	-0.01 (-0.02, -0.001)	**.031**	0.10	-0.01 (-0.03, 0.01)	.07	0.12	-0.01 (-0.03, 0.001)	.05	0.13
Comorbidity	0.05 (-0.01, 0.09)	**.031**	0.11	0.02 (-0.03, 0.07)	.43	0.05	0.02 (-0.03, 0.07)	.51	0.04
Health Condition (*Ref*. *Depression)*									
Epilepsy				0.12 (-0.80, 1.04)	.79	0.02	0.20 (-0.72, 1.12)	.67	0.03
Migraine				0.46 (-1.35, 0.43)	.31	0.08	-0.41 (-1.29, 0.47)	.36	0.07
Multiple Sclerosis				-0.45 (-1.53, 0.62)	.41	0.06	-0.31 (-1.39, 0.77)	.57	0.04
Parkinson Disease				0.46 (-1.39, 0.46)	.33	0.07	-0.35 (-1.27, 0.58)	.46	0.06
Stroke				0.23 (-0.70, 1.16)	.63	0.03	0.32 (-0.61, 1.25)	.50	0.05
Schizophrenia				1.05 (-2.01, -0.01)	.05	0.12	-0.90 (-1.95, 0.15)	.09	0.11
Dementia				-0.08 (-1.05, 0.89)	.87	0.13	-0.10 (-1.05, 0.86)	.84	0.01
Substance Use				-0.04 (-1.99, 0.92)	.94	0.01	0.06 (-0.89, 1.01)	.90	0.01

Note: Model 2 was controlled for health conditions as dummy variables (depression, dementia, epilepsy, migraine, multiple sclerosis, Parkinson’s disease, schizophrenia, stroke and substance use disorders).

*The significant associations are in bold

### Interaction between personality and disability

We additionally assessed in model 3 whether there was a specific interaction between the most important personality and health related factors for social support. The results revealed that the interaction between disability and extraversion was significant (ß = .37, p = .004) as opposed to the interaction between disability and agreeableness (p = .35). However, disability alone remained the most important factor for social support (ß = .66, p = .001) in the model.

### Mediation and Moderation

**Fig 1:**
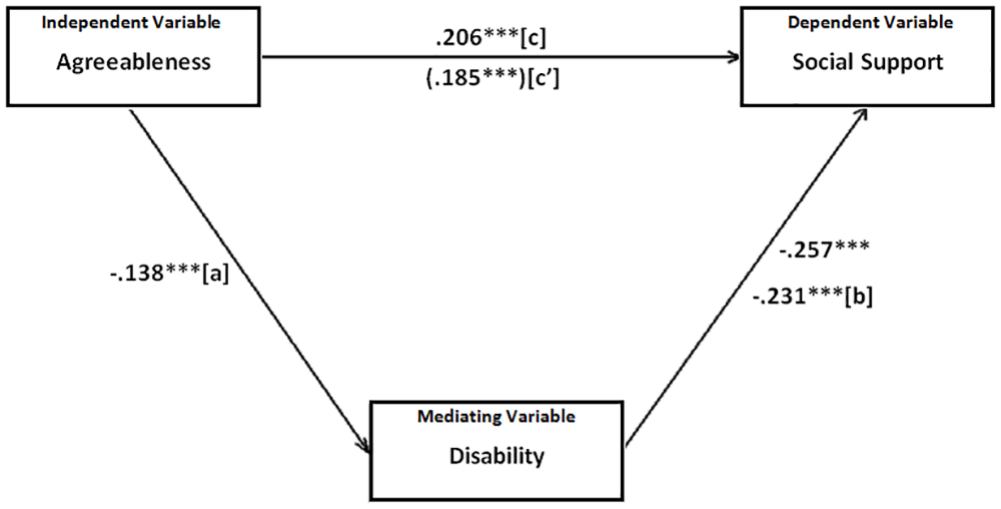
Mediation between agreeableness (IV), disability (MedV) and social support (DV). Note: ***p < .001 The numerical values are zero order correlations. Path c' represents the beta weight for the independent-to-dependent variable relationship adjusted for the inclusion of the mediating variable.

**Fig 2:**
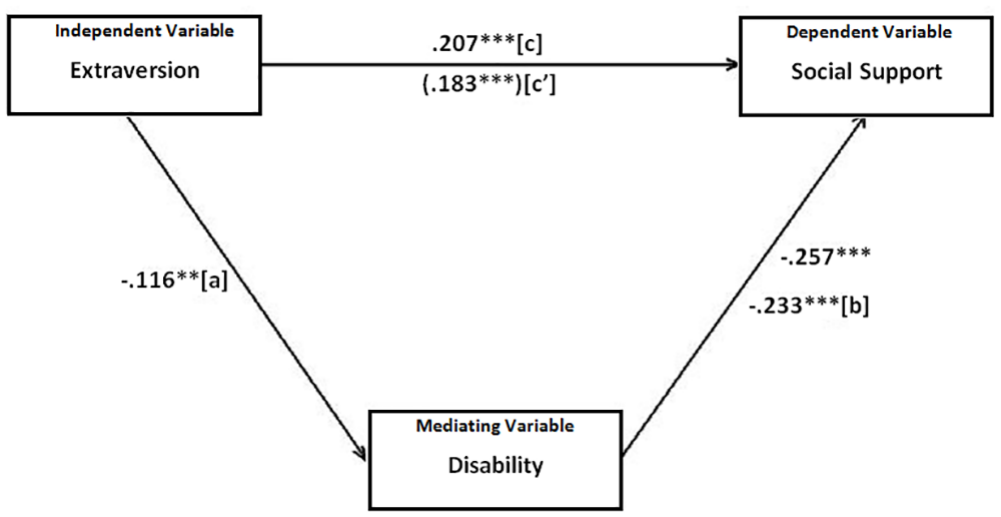
Mediation between extraversion (IV), disability (MedV) and social support (DV). Note: ***p < .001 The numerical values are zero order correlations. Path c' represents the beta weight for the independent-to-dependent relationship adjusted for the inclusion of the mediating variable

Two mediation analyses were conducted including the significant personality traits found in the main multiple regression analysis as independent variables and disability as a mediator. In the first case, presented in Fig 1, disability significantly mediated the relationship between agreeableness and social support (Sobel’s z = 3.18, *p* = .001). The standardized indirect to total ratio was .154, demonstrating that about 15% of the basic relationship between agreeableness and social support was explained partially by the involvement of disability. In the second analysis, shown in Fig 2, a mediation model was tested to determine whether disability significantly mediated the relation between extraversion and social support. The results indicated that disability was a significant mediator (Sobel’s z = 2.77, *p* = .006). The standardized indirect to total ratio was .130, suggesting that about 13% of the link between extraversion and social support can be partially explained by the level of disability of individuals.

**Fig 3:**
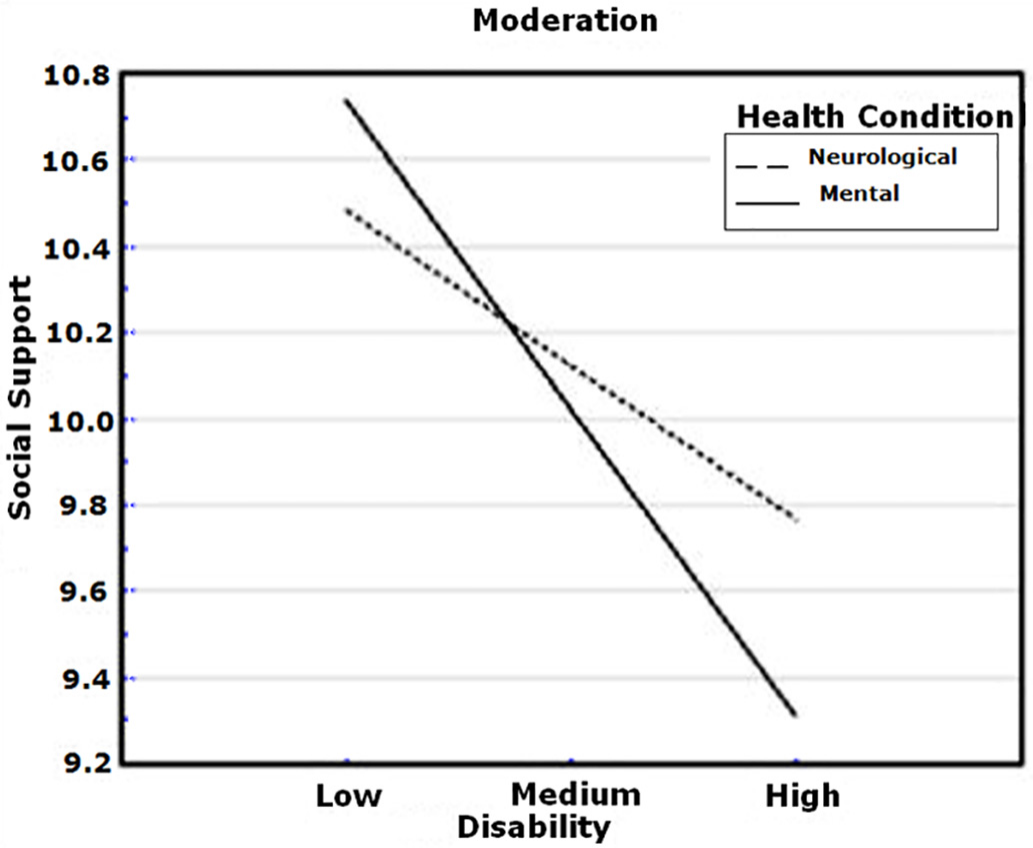
Simple slopes of disability predicting social support for people with mental and neurological health conditions.

Health condition (mental and neurological) was examined as a moderator in the relation between disability and social support. The analysis revealed that it was a significant determinant (interaction effect: ß = .13, *p* = .05). Thus, although higher disability levels were related to lower social support in both groups of health condition, higher levels of disability still decreased the social support in people with mental problems more than in individuals suffering from neurological diseases. Fig 3 shows the simple slopes of the mental and neurological conditions. Both were significantly different from zero (simple slope associated to mental health condition = -0.03, *t* = -5.30, *p* < .001; simple slope associated to neurological health condition = -0.02, *t* = -2.91, *p* = .004).

## Discussion

This study was set to analyze the strongest factors for social support in neuropsychiatric disorders. Disability was the strongest factor associated with social support. As hypothesized, lower levels of disability were strongly associated with higher level of social support. The general direction in previous literature has been towards social support as a predictor or mediator of disability. Yet, the few studies exploring the opposite relationship confirm our finding. Baseman, Fisher [53] found that functional status is a significant predictor of social integration in survivors of stroke. High functional disability has been found to be associated with low social support in depression [16]. Consequently, our results show that the level of social support depends primarily on day-to-day problems that the individuals experience. However, longitudinal research should explore whether the disability leads people to feel they are not supported enough or they feel more disabled because they are not supported.

Disability remained the most significant factor for social support even when the interaction effects with extraversion and agreeableness were added to the model. Extraversion and agreeableness were found to be highly associated with social support and this outcome was consistent with preceding literature [13]. However both variables were no longer significant when the interaction terms were introduced, whilst the interaction term between disability and extraversion was significant.

These results suggest that the interaction between disability and certain personality traits is an important correlate of social support. The cross-sectional design of the study does not allow us to draw causality or explore further the direction of association, but our findings suggest that improving day-to-day functioning and certain maladaptive personality styles may improve individual’s perception of social support and not only vice versa [54]. Literature has already demonstrated that psychological treatment is efficacious in improving functional domains such as interpersonal relationships, social participation, etc.[55, 56], which, in turn, we found to have a strong relationship with social support. On the other hand, modifying certain maladaptive personality styles, reducing Interpersonal Sensitivity or Interpersonal Aggression is possible with long-term therapy (e.g. dialectical behavioral therapy) [57]. Extraversion, for example, which we found to interact with disability, significantly influences character adaptations associated with interpersonal relationships [13]. Thus, clinicians should be aware that modifying certain features of this personality trait might also change the perception of social support by others. Future longitudinal research, however, is warranted to explore in depth this finding.

The present study aimed to investigate the interrelations between personality traits and disability further. The two strongest factors—personality and disability were examined in mediation analyses. The two analyses indicated that disability partially explained the relationship between agreeableness, extraversion, and social support. Extraversion and agreeableness were negatively related to disability, showing that the more agreeable/extraverted a person is the less disability he/she experiences. On other hand, disability negatively predicted social support, indicating that the lower the level of disability is, the higher the social support is. Although the mediations were only partial and other indirect effects could have an empirical importance in the relationship between personality and social support, this outcome implies the importance of disability as a factor that has to be taken into account in future longitudinal analyses.

Finally, the moderation analysis revealed that both sufferers from mental and neurological disorders experienced lower social support when their level of disability was high. In the lower range of disability both mental and neurological disorders experienced similar levels of social support. However, people with mental disorders had lower levels of support compared to neurological disorders in case of high disability. First, this might be because both neurological and mental disorders differ substantially in certain aspects—duration, persistence, recurrence, number of episodes, chronicity. Another possibility may be that mental health problems are socially stigmatized to some extent and severe disability in these health conditions is still not highly recognized as opposed to neurological diseases [19]. The relevance of the finding, although considerable, should be seen cautiously, because of the cross-sectional design of the study.

### Limitations

The study has three main limitations. First, the design is cross-sectional, thus not allowing firm conclusions on causality. The overall sample size, wide scope and innovativeness, yet, do make the findings of the current study relevant. The second limitation is that the data for each disorder was collected in one single country, hence not allowing for more generalizability of the results. This was driven by the fact that the data collection was done according to the expertise of the research centers. Another possible limitation is the use of proxies to obtain data for some of the individuals with dementia. Reports from proxies may have biased the results to some extent in this group. However, 64 individuals were capable of giving their consent and we used proxies for only 16 persons.

## Conclusion

The current paper shed light on an under-researched niche, namely factors for social support in people with neuropsychiatric disorders. Our findings showed that disability was the strongest factor for social support. Extraversion and agreeableness were significant personality variables, but the analyses revealed that they were dependent on the level of disability. Furthermore, findings revealed that the health conditions were not related factors to social support. The study is beneficial for clinicians to tailor support interventions in primary or secondary health care and implement programs encouraging social support in the community. Unlike the previous line of mental health actions, focused on increasing social support as the origin of bettering disability, this study suggests that future interventions should focus effectively on improving disability and maladaptive personality styles as a source of enhancing social support.
